# Supplementary material for: Specific Targeting of Antiapoptotic Bcl-2 Proteins as a Radiosensitizing Approach in Solid Tumors
Source: Int J Mol Sci. 2022 Jul 16;23(14):7850. doi: 10.3390/ijms23147850 (PMC9319836; doi:10.3390/ijms23147850)
Supplement: Supplementary file 1 [file ijms-23-07850-s001.zip › ijms-1746382-supplementary.pdf]

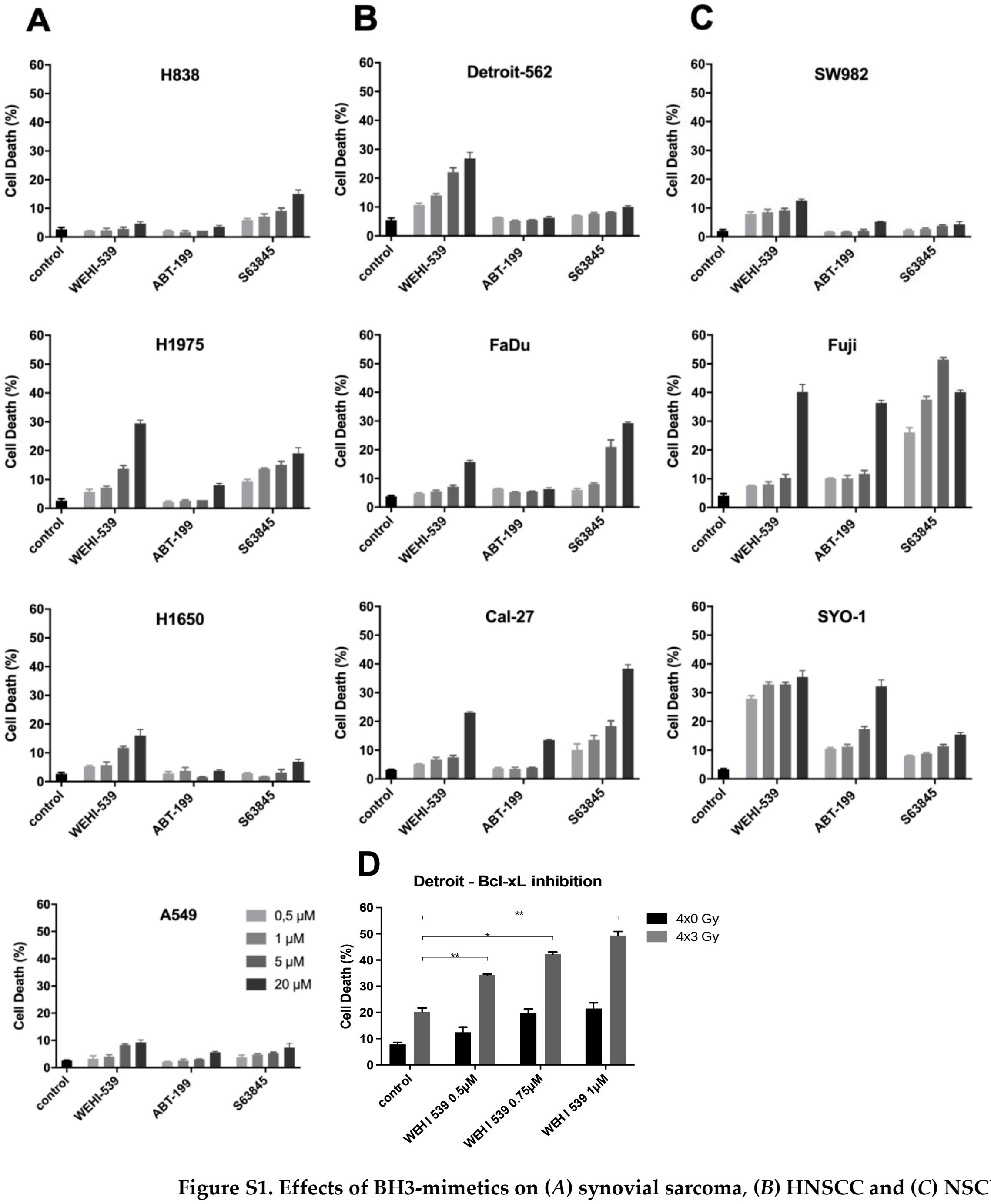

Figure S1. Effects of BH3-mimetics on (*A*) synovial sarcoma, (*B*) HNSCC and (*C*) NSCLC cell lines. FACS-analysis of cell death induction during treatment with BH3-mimetics in different concentrations of 0.5 µM, 1 µM, 5 µM and 20 µM as depicted in the graphic legend. **(*D*)** Detroit cells undergoing fractioned radiation at 4x3 Gy and different concentrations of WEHI-539. Experiments were performed in triplicates and cell death was measured as mean ± SD. The depicted FACS-analyses are representative of at least three independent experiments. $^*p \leq 0.05$, $^{**}p \leq 0.01$.
